# Infrared vibrational spectroscopy: a rapid and novel diagnostic and monitoring tool for cystinuria

**DOI:** 10.1038/srep34737

**Published:** 2016-10-10

**Authors:** Katherine V. Oliver, Annalisa Vilasi, Amandine Maréchal, Shabbir H. Moochhala, Robert J. Unwin, Peter R. Rich

**Affiliations:** 1 Glynn Laboratory of Bioenergetics, Institute of Structural and Molecular Biology, University College London, Gower Street, London WC1E 6BT, United Kingdom; 2 Mass Spectrometry and Proteomics, Institute of Biosciences and Bioresources, National Research Council of Italy, Naples, Italy; 3 UCL Centre for Nephrology, Royal Free Hospital, Pond Street, London NW3 2QG, United Kingdom

## Abstract

Cystinuria is the commonest inherited cause of nephrolithiasis (~1% in adults; ~6% in children) and is the result of impaired cystine reabsorption in the renal proximal tubule. Cystine is poorly soluble in urine with a solubility of ~1 mM and can readily form microcrystals that lead to cystine stone formation, especially at low urine pH. Diagnosis of cystinuria is made typically by ion-exchange chromatography (IEC) detection and quantitation, which is slow, laboursome and costly. More rapid and frequent monitoring of urinary cystine concentration would significantly improve the diagnosis and clinical management of cystinuria. We used attenuated total reflection - Fourier transform infrared spectroscopy (ATR-FTIR) to detect and quantitate insoluble cystine in 22 cystinuric and 5 healthy control urine samples. Creatinine concentration was also determined by ATR-FTIR to adjust for urinary concentration/dilution. Urine was centrifuged, the insoluble fraction re-suspended in 5 μL water and dried on the ATR prism. Cystine was quantitated using its 1296 cm^−1^ absorption band and levels matched with parallel measurements made using IEC. ATR-FTIR afforded a rapid and inexpensive method of detecting and quantitating insoluble urinary cystine. This proof-of-concept study provides a basis for developing a high-throughput, cost-effective diagnostic method for cystinuria, and for point-of-care clinical monitoring

Cystinuria is an autosomal inherited aminoaciduria caused by mutations in one or both subunits of the amino acid transport system b^0,+^, and has a global prevalence ranging from 1 in 2000 to 1 in 100000, depending on the population^1^. It results in a failure to reabsorb freely filtered cystine in the proximal tubule of the kidney, causing increased urinary excretion of cystine and the dibasic amino acids; lysine, arginine and ornithine^2^. Cystine is a dimer of cysteine formed by oxidation of their sulphydryl groups to form a disulphide bond. It is poorly soluble in water in the physiological pH range^3^. Its solubility limit in urine is ~1 mM^1^,^4^ and it forms a microcrystalline precipitate above this limit. This insoluble cystine can form recurrent stones that may cause kidney obstruction and lead eventually to irreversible damage and loss of function. Total cystine levels in cystinuric patients typically range from 1 to 2 mM, but concentrations over 4 mM have been reported^5^. Patients with urinary cystine concentrations above the solubility limit have a significantly increased risk of stone formation^1^,^2^. Many patients respond to simple measures such as increased fluid intake, urinary alkalinisation, an alkaline ash or low methionine (animal protein) diet, and dietary salt restriction^6^. Dithiol drugs can also decrease urinary cystine by disulphide bond reduction and formation of more soluble drug-cysteine complexes. However, these measures do not treat the underlying cause of the disease and are most effective when used prophylactically^1^. Once stone formation has occurred, shock-wave lithotripsy or surgery is usually required.

Cystinuria is commonly diagnosed by measuring urinary cystine^1^. Early diagnosis and preventive maintenance therapy are essential and require a means to detect and quantitate cystine repeatedly. Qualitative colorimetric screening tests are available, but these have low specificity^7^,^8^. Derivatisation followed by ion exchange chromatography (IEC) is the current clinical gold standard for amino acid analyses, including cystine^7^. However, IEC is time-consuming and expensive in personnel and equipment. As a result, it is not generally available at the point-of-care. Alternatively, urinary cystine solubility threshold can be quantitated by a cystine capacity assay^9^, but again this is labour-intensive and not widely available. A faster, simpler and more cost-effective routine method for quantitating urinary cystine would be of significant clinical value for both diagnosis and monitoring of cystinuria.

Fourier transform infrared (FTIR) vibrational spectroscopy is a powerful chemical analytical technique. It can provide rapid, quantitative and reproducible analyses of multiple components in complex mixtures^10^ and thus provides an alternative means for quantitative evaluation of some clinical biomarkers. Data collection has been greatly simplified with robust attenuated total reflection (ATR) microprisms that allow FTIR spectra to be obtained simply by placing the test material on the surface of the ATR prism^11^. Many types of molecule are infrared active and have unique patterns of absorbance; hence, IR spectra of biological materials are potentially rich in information, but can be complex and difficult to deconvolute. IR analysis of the composition of kidney stones is already used clinically^12^,^13^,^14^ and has been applied to cystine stones^15^. There is also a rapidly expanding literature on analyses of levels of specific constituents in complex biological fluids such as urine^16^,^17^,^18^,^19^,^20^ and blood^16^,^21^,^22^ and, with 2D imaging methods, mapping of different cell types in normal and diseased tissue sections^23^,^24^,^25^,^26^,^27^, including kidney^28^,^29^. In this report we explore the potential of ATR-FTIR spectroscopy as a rapid, reagent-free clinical method for detecting and quantitating insoluble cystine in urine.

## Results

### Quantitation of urea and creatinine in urine by ATR-FTIR spectroscopy

Variations in both fluid intake and glomerular filtration rate (GFR) affect the concentrations of materials excreted in urine. Creatinine is a breakdown product of creatine phosphate in muscle and is normally formed at a fairly constant rate that is dependent on muscle mass. Hence, its concentration in urine is a good indicator of overall urine dilution and concentrations of other components are commonly factored by creatinine concentration to allow for urinary concentration/dilution.

Urinary creatinine and urea were determined by deconvolution of the 1510–1445 cm^−1^ region of undried urine FTIR absorbance spectra as described in Methods. Figure S3 shows an example of the deconvolution and of a typical ATR-FTIR spectrum of urine from a cystinuric patient. Figure S4 summarises the urea and creatinine concentrations determined by this method in urine samples from 22 cystinuric patients (labelled from P1 to P24) and 5 healthy controls. Urea concentrations were 28–443 mM and creatinine concentrations were <1–20 mM, values within the typical range. As expected, the concentrations of urea and creatinine tend to correlate and no ratio difference between cystinuric and healthy samples was evident.

### Comparison of ATR-FTIR and Jaffe methods of creatinine quantitation

**Figure 1:**
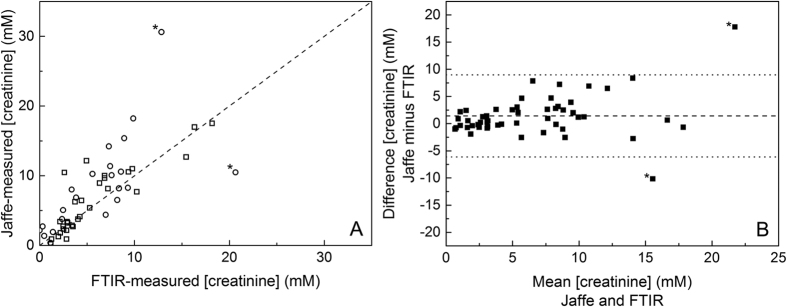
Comparison of the Jaffe and ATR-FTIR methods of creatinine quantitation. (**A**) Plot of urinary creatinine concentrations determined by the Jaffe (Y axis) and ATR-FTIR (X axis) methods. (**B**) Bland-Altman plot of the same data. Circles: samples for which cystine content was also determined; squares: additional urine samples.

To validate the ATR-FTIR method for urinary creatinine quantitation, creatinine concentrations in the same urine samples were also determined with the Jaffe reaction, the most widely used clinical method^30^,^31^. A reasonable correlation was observed between the two methods (Fig. 1A) with a Pearson correlation coefficient, r, of 0.75, despite two outliers (indicated with stars). A Bland-Altman analysis^32^ was also performed to establish the similarity between the results generated by the two methods (Fig. 1B). The dashed line shows the mean bias and the dotted lines show 95% confidence limits. The mean bias was 1.4 mM, demonstrating that the Jaffe reaction tended to indicate higher creatinine concentrations. All data points apart from the two outliers fell within the 95% confidence limits. The origin of the differences in concentrations predicted by the Jaffe and ATR-FTIR methods in the two outliers was not investigated further. However, for the most extreme outlier, the 30 mM value determined by the Jaffe reaction (12.5 mM with ATR-FTIR) appeared to be much higher than expected given the 275 mM urea concentration in this same urine sample.

### Quantitation of cystine by ATR-FTIR spectroscopy

**Figure 2:**
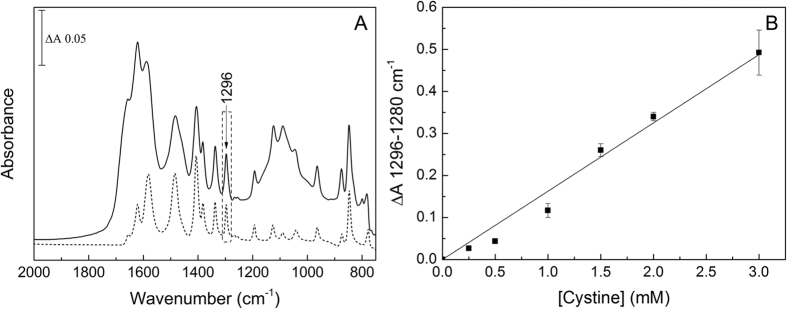
Absorbance spectra of cystine and insoluble cystinuric urine. (**A**) Dry cystine (dashed line) and a dried insoluble fraction of a cystinuric urine sample (solid line). (**B**) Calibration curve for pure cystine based on the differences in intensities at 1296 cm^−1^ minus 1280 cm^−1^ of their second derivative spectra. A linear fit through the origin was calculated with no error weighting.

The ATR-FTIR spectrum of dried L-cystine is shown in Fig. 2A. A calibration plot (Fig. 2B) was generated from differences in intensities at 1296 minus 1280 cm^−1^ of the second derivatives of absorbance spectra of dried 5 μL aliquots of 0 to 3 mM cystine suspensions. A linear line of best fit through the origin was a reasonable fit in this concentration range, confirming that even the highest cystine levels result in a layer that remains within the active spectroscopic volume delineated by the depth of penetration of the evanescent wave^11^. This is consistent with the cystine molecular volume of 193 Å^3^ and an active spectroscopic volume of height ~1 micron and diameter 3 mm, which would accommodate ~60 nmoles cystine (i.e. equivalent to 5 μL of a 12 mM cystine suspension).

Although drying of urine samples onto the ATR prism considerably increases the intensities of absorbance bands of cystine, absorbance bands of other components will also be amplified. These could mask some cystine bands, or might increase the layer thickness beyond the evanescent wave limit. Because of this, and because the clinically important parameter is the insoluble cystine, a protocol was developed which measured the insoluble components after removal of the majority of soluble material. This was achieved by centrifugation of urine samples to form a pellet of the insoluble components that could be separated from supernatant that contained the solutes. Pellets were resuspended in water and aliquots were dried onto the prism and absorbance spectra were recorded. The characteristic band pattern of cystine (Fig. 2A, dashed line) could clearly be seen in the spectra of most of the dried, insoluble fractions derived from cystinuric patient urine (Fig. 2A, solid line) and could be quantitated using the calibration plot (Fig. 2B). Hence, this establishes proof of concept that insoluble cystine at levels present in cystinuric patient urine samples can indeed be detected and quantitated using ATR-FTIR technology.

**Figure 3:**
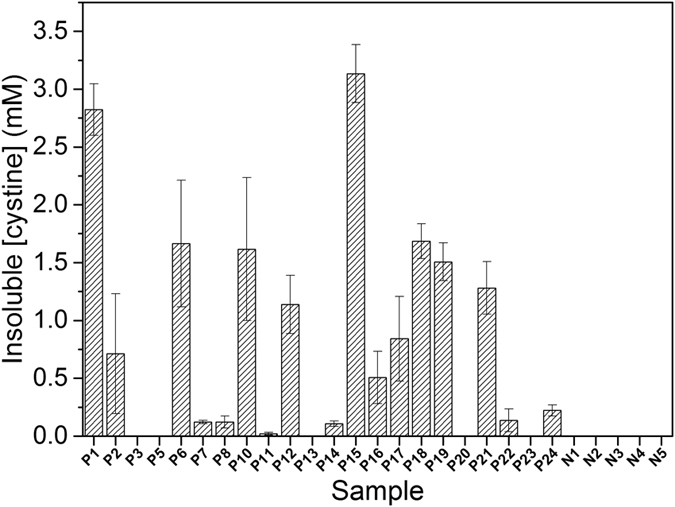
Insoluble cystine in urine samples determined by ATR-FTIR spectroscopy. Insoluble cystine was determined by the ATR-FTIR method described in the text in cystinuric (*Pn)* and control (*Nn*) urine samples. Samples were measured in triplicate and error bars represent standard error of mean.

**Figure 4:**
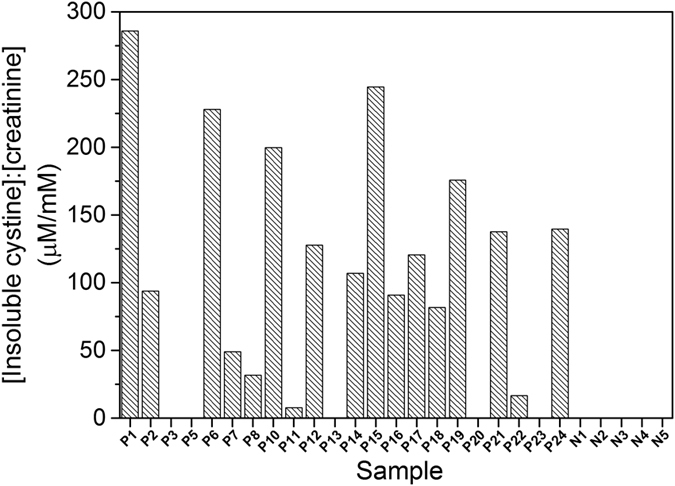
Insoluble cystine:creatinine ratios in urine determined by ATR-FTIR spectroscopy. Urinary cystine:creatinine ratios (μM:mM) were calculated from creatinine and insoluble cystine levels determined by the ATR-FTIR methods described in the text in cystinuric (*Pn)* and control (*Nn*) urine samples. Samples were measured in triplicate and error bars represent standard error of mean.

The concentrations of insoluble cystine in 22 cystinuric and 5 healthy control patient urine samples were analysed (Fig. 3) using this protocol. Seventeen of the cystinuric patient samples had detectable insoluble cystine of up to 3.1 mM. Five cystinuric patient samples and all healthy controls had no detectable insoluble cystine, indicating that their urinary cystine concentrations were below the solubility limit of ~1 mM. In order to accommodate variations in urine concentrations, the insoluble cystine values were also ratioed to the ATR-FTIR-determined creatinine concentrations (Fig. 4).

### Comparison of FTIR and clinical methods of cystine quantitation

**Figure 5:**
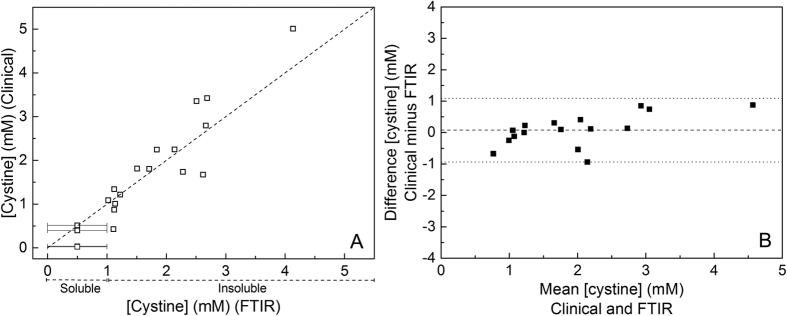
Comparison of the ion exchange chromatographic and ATR-FTIR methods of cystine quantitation. (**A**) Plot of urinary cystine concentrations determined by the IEC (Y axis) and ATR-FTIR (X axis) methods. (**B**) Bland-Altman plot of the same data. A 1 mM offset has been added to the ATR-FTIR values to account for the soluble cystine since it measures only the insoluble cystine (see text for details).

The cystine concentrations determined by ATR-FTIR spectroscopy were compared with those obtained by the clinical gold standard of ion exchange chromatography after derivatisation. The ATR-FTIR method measures only insoluble cystine, which is the clinically relevant fraction, whilst the clinical method reports total (soluble + insoluble cystine). Hence, for a comparison of the total cystine obtained by the two methods, an offset of 1 mM (solubility limit for cystine) was added to those values obtained by ATR-FTIR that had measureable insoluble cystine to allow for the ~1 mM soluble cystine fraction that must have been present in the original urine samples. For samples which showed no ATR-FTIR-detected insoluble cystine, their cystine must have been below the 1 mM solubility limit and so have been plotted as 0.5 ± 0.5 mM. Figure 5A shows that there was a good correlation between the results obtained by the two methods (Pearson correlation coefficient, r, of 0.915). A Bland-Altman analysis^32^ of the same data (Fig. 5B) indicated little or no bias between the methods and data points fell within the 95% confidence limits.

## Discussion

The purpose of this study was to assess whether ATR-FTIR spectroscopy could provide a novel quantitative method for easier cystinuria diagnosis and routine monitoring of urinary cystine that is fast and cost-effective in comparison with the ‘gold-standard’ of IEC. We focused on the quantitation of the insoluble cystine fraction, which can easily be separated from possible interfering soluble components, because it is this insoluble material that promotes stone formation and disease complications in cystinuric patients.

To allow for large differences in urine sample dilutions, the insoluble cystine concentrations were ratioed to urinary creatinine concentrations that were also determined by ATR-FTIR spectroscopy. These ATR-FTIR-derived creatinine concentrations were validated by comparison with values obtained by the clinical standard Jaffe reaction. A reasonable correlation between the two methods was found, though the Jaffe reaction tended to give slightly higher values. This could be a result of additional chromogens in urine that interfere with the Jaffe method^30^,^33^,^34^. For all but two samples, the concentrations derived by the two methods fell within the 95% confidence interval^32^.

Sufficiently accurate quantitation of the insoluble urinary cystine was also achieved by ATR-FTIR spectroscopy, using intensities at 1296 minus 1280 cm^−1^ in the second derivative spectra of the dried, insoluble fractions derived from the urine samples. These wavenumbers were chosen because there is no other strong contributor from other insoluble components (for example, oxalate, urate and dihydroxyadenine) that might be present in urine from patients with different kidney stone-related conditions. A linear line through the origin (Fig. 2b) of a calibration plot with pure cystine was a reasonable fit to at least 3 mM cystine, which covers the typical range of the cystinuric urine samples. The cystine levels measured by the ATR-FTIR spectroscopy were validated by parallel IEC measurements. However, the FTIR protocol described here measures only the insoluble cystine, whilst the IEC method measures total cystine without specifying soluble and insoluble fractions. Hence, for comparison of the two methods, a nominal 1 mM soluble cystine was added to the IR-determined insoluble cystine values in order to allow direct comparison to the IEC-determined total (soluble plus insoluble) cystine estimates.

Consistent with the IEC measurements, the ATR-FTIR method revealed that 17 of the 22 cystinuric patient samples had urinary cystine above its solubility threshold, whereas none of the control samples has detectable insoluble cystine. Since all of the cystinuric patients included in this study were being advised on their fluid intake and diet, and some were receiving drug treatment (Table S1), it could be expected that this management may have resulted in clearance of insoluble cystine in some instances. Indeed, four of the five cystinuric patients with no FTIR-detectable insoluble cystine (P5, P13, P20 and P23) were receiving cystine-decreasing therapies. A fifth, P3, also had no FTIR-detectable insoluble cystine, but was not on drug treatment and the creatinine concentration was not particularly low, suggesting that this patient suffered from only mild cystinuria. Of the six cystinuric patients with relatively low (<0.5 mM) FTIR-detected insoluble cystine (P7, P8, P11), three were also receiving cystine-decreasing therapies; two also (P14 and 24) had low creatinine suggesting dilute urine samples; one (P22) had low insoluble urinary cystine with moderate/high creatinine despite no drug treatment. In contrast, high levels of cystine were recorded in P1, P6, P15, P18 and P19; these patients were either not receiving drug treatment for cystinuria (P6, P15 and P19) and/or had concentrated urine as judged by relatively high creatinine concentrations (P1, P15 and P18). The above considerations emphasise the point that, when assessing stone formation risk from these data, a more useful clinical parameter may be the cystine:creatinine (Fig. 4). For example, P1 and P18 had comparatively high insoluble cystine concentrations, but P1 had a much higher insoluble cystine:creatinine ratio, suggesting that this patient might have a more severe form of cystinuria. Similarly, although P14 had relatively low insoluble cystine, the cystine:creatinine ratio suggested that the level could be of more serious concern. It might be emphasised that essentially the same diagnoses result from the more time-consuming and expensive IEC analyses of the same samples.

In summary, we demonstrate proof-of-concept of a simple FTIR method to detect insoluble cystine levels across the mild to severe levels that are found in most cystinuric patients, based on its 1296 cm^−1^ absorbance band in spectra of dried insoluble fractions of urine samples. Although the FTIR method provides an estimate for insoluble cystine that is comparable to data obtained by the IEC clinical standard method, it must be emphasised that the protocol as described provides a measure only of insoluble cystine, whereas the IEC method provides quantitative information on total cystine, together with a wide range of amino acids. Nevertheless, the cost of suitable equipment for the FTIR method is considerably less than that required for IEC, especially if the processing time and labour costs are included. Furthermore, the FTIR method requires minimal expertise (no formal laboratory training, as required for IEC) and no chemical manipulations or expensive consumable reagents or materials. These factors, combined with the possibility of rapid (and potentially ‘at the bedside’) throughput with modern room-temperature ATR-FTIR spectrometers, means that the FTIR method could provide a considerably more cost-effective means of urinary cystine detection (initial diagnosis) and quantitation (clinical management). This could allow more widespread patient screening for cystinuria and, of importance, provides a cost-effective means for frequent monitoring of insoluble patient urinary cystine variations during treatment, facilitating accurate assessment of disease severity and responses to treatment. Further enhancements of sensitivity and specificity could be achieved by inclusion of additional cystine bands (e.g. those at 845 and 775 cm^−1^) in the analytical algorithm or by more advanced quantitation methods using, for example, principal component analysis (PCA). Protocols could also be adapted for use with a high-throughput, automated transmission mode FTIR spectrometer equipped with multi-sample silicon plates and extension to other stone-associated insoluble compounds such as oxalate and urate.

## Methods

### Patients and Materials

The study was performed in accordance with the relevant guidelines and regulations for studies in human subjects and was approved by the Royal Free London NHS Foundation Trust Ethics Committee (R&D ref: 7727; REC number: 05/Q0508/6). Urine samples were collected in the outpatient clinic, as they are routinely, as mid-stream urine collections after obtaining informed consent for further research analysis, in addition to any routine clinical care analyses, from patients attending the Royal Free Hospital Metabolic Stone and Renal Service, London, UK; samples were stored at −20 °C. Urine samples were obtained from 22 cystinuric patients, 5 healthy volunteer donors as controls, and (for creatinine analysis only) 11 additional patients with other renal conditions. All chemicals used were obtained from Sigma-Aldrich, Dorset, UK.

### ATR-FTIR spectroscopy

Data were recorded with a dry air-purged Bruker IFS 66/S FTIR spectrometer operated with Bruker OPUS 6.5 software and fitted with a liquid nitrogen-cooled MCT-A detector, a KBr beam splitter and an ATR microprism (SensIR; 3-reflection silicon microprism with ZnSe optics). IR spectra were recorded at room temperature between 4000–750 cm^−1^. Typically, 500 interferograms at 4 cm^−1^ resolution were averaged (approx. 60s acquisition time) before Fourier transformation. Absorbance spectra were computed *versus* a background power spectrum of the clean prism surface. Frequencies quoted have an accuracy of ~±1 cm^−1^. Spectral contributions from water vapour in the optical path and (for samples in aqueous media) liquid water were removed by fractional subtraction of pure water vapour and liquid water reference spectra, respectively, with OPUS 6.5 software.

### Quantitation of urea and creatinine by FTIR spectroscopy

Creatinine can be determined from IR spectra of whole urine with various deconvolution methods^16^,^17^,^18^,^19^,^35^. In the present study urea and creatinine were quantitated using the 1510–1445 cm^−1^ region of ATR-FTIR absorbance spectra of ‘as-collected’, undried urine since this region is dominated by urea and creatinine contributions^35^. For comparison, urinary creatinine was also assayed with the Jaffe reaction^36^, a protocol used in standard clinical practice. Details of methods are provided in Supplementary Information.

### Quantitation of insoluble urinary cystine by ATR-FTIR spectroscopy

In order to increase the sensitivity of cystine detection to those relevant to cystinuria (several mM), a quantitative protocol was developed. Samples were dried onto the prism surface to form layers sufficiently thin that they did not fill the spectroscopically-active volume above the prism surface. A calibration plot was created by drying 5 μL aliquots of 0 to 3 mM total (soluble + insoluble) L-cystine and measuring differences in intensities between 1296 cm^−1^ and 1280 cm^−1^ of their second derivative spectra (details in Supplementary Information). A linear line of best fit through the origin with no error weighting was calculated using Origin 8.5 software (OriginLab, Northampton, MA, USA) for use as a calibration plot for urinary cystine concentrations.

Insoluble urinary material including cystine was separated from urea, creatinine and other solutes by centrifugation of 1 mL aliquots of the urine samples at 16000 g_av_ for 40 minutes. Pellets were re-suspended in 1 mL double distilled water and thoroughly mixed by vortexing. 5 μL aliquots were dried onto the prism surface and second derivative forms of absorbance spectra were recorded as described above. Insoluble cystine concentrations in the original urine samples were determined from the differences in intensities between 1296 and 1280 cm^−1^ in relation to the calibration plot. For validation, cystine concentrations in the same urine samples were also determined by IEC, the gold standard in clinical amino analysis^7^. Details are provided in Supplementary Information.

### FTIR data validation

Bland-Altman plots^32^ were used to compare both the FTIR and Jaffe methods for quantitating creatinine, and the FTIR and IEC methods for quantitating cystine. The mean bias for each plot was calculated and plotted as a dashed line and 95% confidence limits (mean bias +/−1.96 standard deviations) are indicated by dotted lines.

## Additional Information

**How to cite this article**: Oliver, K. V. *et al*. Infrared vibrational spectroscopy: a rapid and novel diagnostic and monitoring tool for cystinuria. *Sci. Rep.* **6**, 34737; doi: 10.1038/srep34737 (2016).

## Supplementary Material

Supplementary Information
