# Supplementary material for: Infrared vibrational spectroscopy: a rapid and novel diagnostic and monitoring tool for cystinuria
Source: Sci Rep. 2016 Oct 10;6:34737. doi: 10.1038/srep34737 (PMC5056377; doi:10.1038/srep34737)
Supplement: Supplementary Information [file srep34737-s1.pdf]

Infrared vibrational spectroscopy: a rapid and novel diagnostic and monitoring tool for cystinuria

Katherine V Oliver, Annalisa Vilasi, Amandine Maréchal, Shabbir H Moochhala, Robert J Unwin, Peter R Rich

Supplementary Information

Quantitation of urea and creatinine by FTIR spectroscopy

Creatinine can be determined from IR spectra of whole urine with various deconvolution methods [1-5]. In the present study urea and creatinine were quantitated using the 1510-1445 cm^{-1} region of ATR-FTIR absorbance spectra of 'as-collected', undried urine since this region is dominated by urea and creatinine contributions [5]. Optimal parameters for component fitting were determined from spectra of solutions of pure urea and creatinine in double distilled water (**Figures S1a, S1b**) at concentrations typically found in urine. In all cases, 20 µL aliquots were placed on the prism, which was sufficient to fill the entire spectroscopically-active volume above the prism surface. The urea spectrum in this region can be reasonably fitted with a single Gaussian component centred at 1468 cm^{-1} with full width at half maximum (FWHM) of 32 cm^{-1} and the creatinine spectrum with a single Lorentzian component centred at 1492 cm^{-1} with a FWHM of 24 cm^{-1} (for a more complex deconvolution of this region required for dried and partially hydrated states, see [5]). The best fits of these two components, together with an unrestricted linear baseline, were made to spectra of solutions of pure compounds and mixtures using the 'Peak

Analyzer' function of Origin 8.5 software. Calibration curves were generated from intergrals of peak areas *versus* urea or creatinine concentrations using solutions of urea ranging from 25 mM to 500 mM and of creatinine from 2 mM to 30 mM (**Figure S2**). The method was validated by application to quantitation of urea and creatinine in known mixtures at typical urinary concentrations and had an accuracy of $\pm$5% (**Figure S1c, S1d**). The same parameters and protocols were used to determine urinary urea and creatinine concentrations from absorbance spectra of 20 µL aliquots of 'as collected', undried urine samples (**Figure S4)**.

Quantitation of creatinine by the Jaffe reaction

For comparison, urinary creatinine was also assayed with the Jaffe reaction [6], a protocol used in standard clinical practice. The Jaffe working reagent was prepared by mixing five parts of 200 mM NaOH with one part of 40 mM picric acid. Absorbance at 510 nm was monitored using a Shimadzu 50000 UV visible spectrophotometer at 25°C. 0-30 mM creatinine standards were diluted 50-fold in water. 3 mL of working reagent were placed in a glass cuvette and the rate of change of absorbance at 510 nm was recorded on addition of 200 µL diluted creatinine. Initial rates of reaction were plotted *versus* creatinine concentrations to generate a calibration curve. The same protocol was used for estimations of creatinine concentrations in urine samples.

Quantitation of cystine by ATR-FTIR spectroscopy

In order to increase the sensitivity of cystine detection to those relevant to cystinuria (several mM), a quantitative protocol was developed in which samples were dried onto the prism surface to form layers sufficiently thin that they did not fill the spectroscopically-active volume above the prism surface. 111 mg of L-cystine was first dissolved in 2 mL of 1 M HCl. Ten mL of a 50 mM solution of sodium phosphate

was then added followed by 2 mL of 1 M NaOH, generating a very fine cystine precipitate. The suspension was adjusted to a pH of 7 with NaOH. This stock suspension of 32 mM total (soluble + insoluble) cystine was mixed by vortexing before dilution with double distilled water to prepare samples of 0 to 3 mM total cystine. 5 µL aliquots were dried onto the prism surface under a gentle stream of dry nitrogen gas (flow rate of 300 mL/min). Once dried, even pressure was applied to the sample using a standard press (SensIR) to flatten the sample and ensure good contact between the relatively hydrophilic cystine and the hydrophobic surface of the ATR prism. Absorbance spectra were recorded and converted to their second derivative forms after smoothing using the Savitzky-Golay algorithm with a 17 point window. The differences in intensities between 1296 cm^{-1} and 1280 cm^{-1} were plotted *versus* total cystine concentrations of the original suspensions. This 1296 cm^{-1} band was chosen because it is in a region that is relatively free from overlapping bands of other urinary components. A linear line of best fit through the origin with no error weighting was used as a calibration for urinary cystine concentrations.

Quantitation of insoluble urinary cystine by FTIR spectroscopy

Insoluble urinary material including cystine was separated from urea, creatinine and other solutes by centrifugation of 1 mL aliquots of the urine samples at 16000 g_{av} for 40 minutes. Pellets were resuspended in 1 mL double distilled water and thoroughly mixed by vortexing. 5 µL aliquots were dried onto the prism surface and FTIR absorbance spectra were recorded as described above. Absorbance spectra were converted to their second derivative forms after smoothing using the Savitzky-Golay algorithm with a 17 point window. Insoluble cystine concentrations in the original urine samples were determined from the differences in intensities between 1296 and 1280 cm^{-1} in relation to the calibration plot.

Quantitation of urinary cystine by ion exchange chromatography

Cystine concentrations were determined by IEC, the gold standard in clinical amino analysis [7], using a Biochrom 30+ Amino Acid Analyzer by staff at the Camelia Botnar Laboratories, Great Ormond Street Hospital. Samples were warmed to room temperature, deproteinised with sulphosalicylic acid containing norleucine as an internal standard, and loaded onto a column linked to sulphonate groups to permit charge-charge interaction. Amino acids were sequentially eluted over 2-3 hours with increases in temperature, pH and ionic strength using lithium citrate.

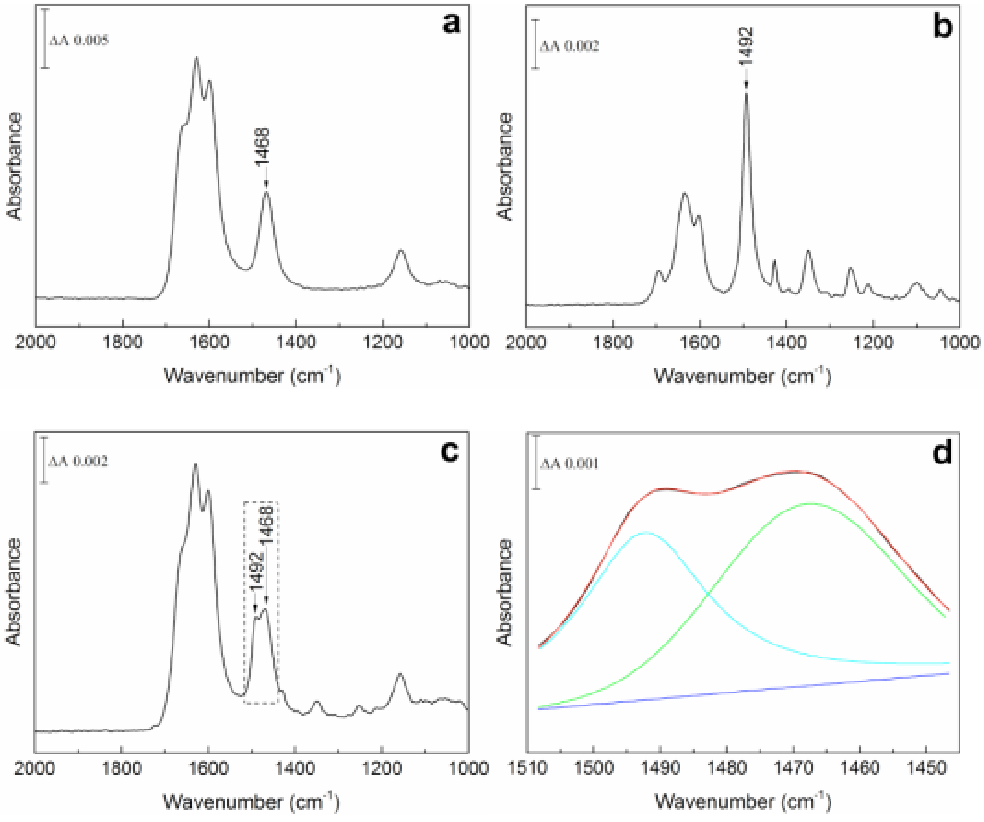

Figure S1. ATR-FTIR spectra of pure urea and creatinine in aqueous solution are shown in **Figure 1a** and **b**, respectively. Urea or creatinine components were fitted to the region between 1510 cm^{-1} and 1445 cm^{-1} This method was validated by application to the ATR-FTIR spectrum of a mixture of 100 mM urea and 10 mM creatinine in water (**Figure 1c**), giving values of 100 mM (± 1 mM) and 10.6 mM (±0.5 mM), respectively (**Figure 1d**).

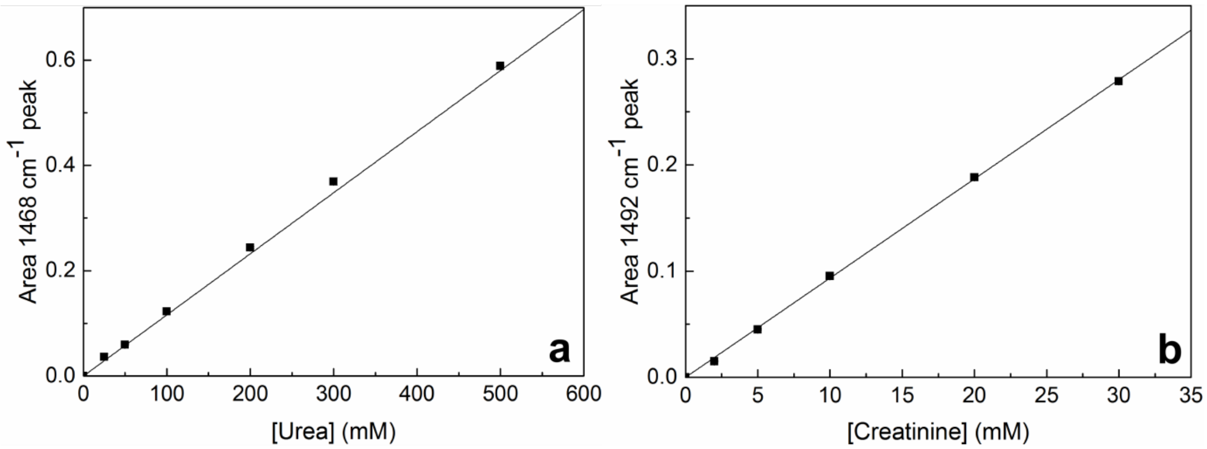

Figure S2. Calibration curves for urea and creatinine in water. ATR-FTIR spectra of aqueous urea and creatinine solutions at different concentrations were recorded. After subtraction of water contributions, components were fitted between 1510 cm^{-1} and 1445 cm^{-1}. (**a**) Area of the 1468 cm^{-1} component versus urea concentrations; (**b**) area of the 1492 cm^{-1} component versus creatinine concentrations.

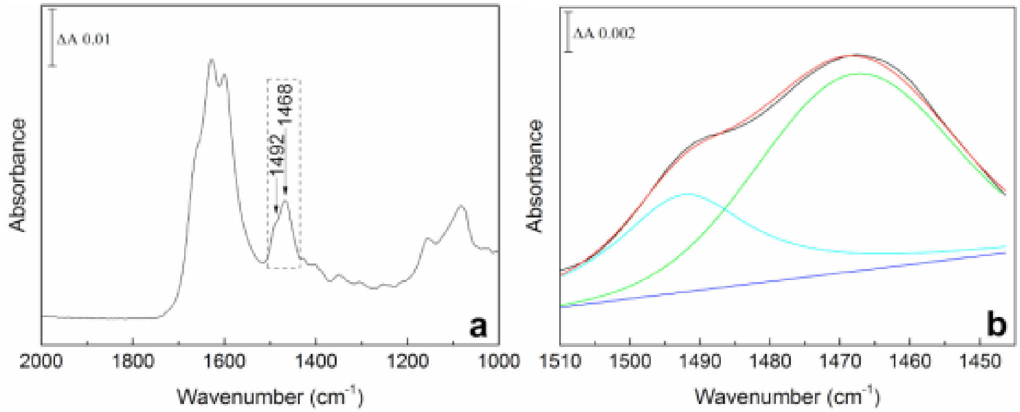

Figure S3. Deconvolution of urea and creatinine in a typical urine sample. (a) Spectra of a typical ‘as collected’ urine sample after subtraction of contributions of water solvent. (b) deconvolution of the 1510-1445 cm^{-1} region into creatinine (light blue), urea (green) and linear baseline (dark blue). The data are shown in black and the overall simulation fit is in red.

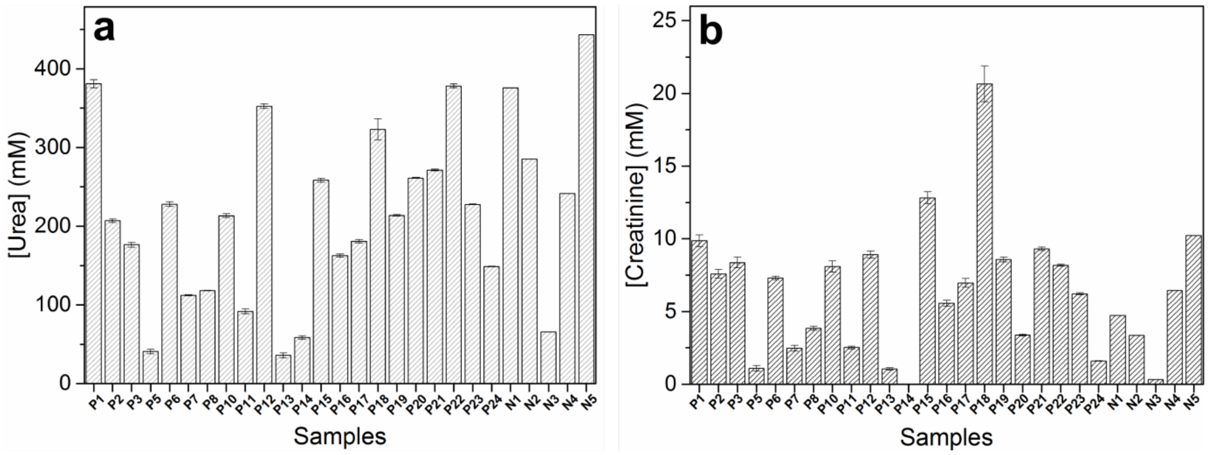

Figure S4. Urea and creatinine concentrations in whole urine determined from ATR-FTIR spectra. Concentrations were determined as described in Methods: P*n*: cystinuric patient urine; N*n*: healthy control urine. Samples were measured in triplicate and error bars represent standard error of mean.

	Age (years)	Sex	Current cystinuria treatment	Mean [Creatinine] (mM)	Mean [Cystine] (mM)	Cystine : creatinine (μM/mM)
P1	19	Female	Potassium citrate (1 tablet bd)	9.9	2.82	285
P2	22	Male	None	7.6	0.71	94
P3	33	Male	None	8.4	0.00	0
P5	36	Female	Tiopronin (250 mg tds)	1.1	0.00	0
P6	22	Male	None	7.3	1.66	228
P7	21	Male	Tiopronin (250 mg tds)<br>Potassium citrate (2 tablets qds)<br>Effercitrate (2 tablets qds)	2.5	0.12	49
P8	54	Female	Penicillamine (750 g nocte)<br>Penicillamine (250 mg mane)	3.8	0.12	32
P10	29	Male	Potassium citrate (1 tablet bd)<br>Tiopronin (500 mg nocte and 250 mg mane)	8.1	1.62	200
P11	59	Female	Tiopronin (250 mg bd)	2.5	0.02	8
P12	64	Male	Potassium citrate (10 ml tds)	8.9	1.14	128
P13	32	Female	Potassium citrate (1 tablet bd)	1.0	0.00	0
P14	59	Male	None	0.0	0.11	107
P15	35	Female	None	12.8	3.13	245
P16	36	Female	Tiopronin (250 mg tds)	5.6	0.51	91
P17	22	Male	Potassium citrate (1 tablet bd)<br>Tiopronin (200 mg bd)	7.0	0.84	121
P18	67	Female	Tiopronin (500 mg bd)<br>Potassium citrate (2 tablets bd)	20.6	1.69	82
P19	34	Female	None	8.6	1.51	176
P20	48	Female	Sodium bicarbonate (1 g nocte)<br>Penicillamine (500 mg od)	3.4	0.00	0
P21	52	Male	Potassium citrate (10 ml tds)	9.3	1.28	138
P22	54	Male	None	8.2	0.14	17
P23	63	Female	Penicillamine (250 mg tds)	6.2	0.00	0
P24	34	Female	None	1.6	0.22	140

Supplementary Table 1. Data on cystinuric patients used in study. Cystine and creatinine data were determined by ATR-FTIR methods (od, once daily; bd, twice daily; tds, thrice daily; qds, four times per day; nocte, at night;, mane, in morning).

References

1. Hoşafçi, G., Klein, O., Oremek, G. & Mäntele, W. Clinical chemistry without reagents? An infrared spectroscopic technique for determination of clinically relevant constituents of body fluids. *Anal Bioanal Chem* **387,** 1815–1822 (2007).
2. Shaw, R., Low-Ying, S., Leroux, M. & Mantsch, H. Toward reagent-free clinical analysis: quantitation of urine urea, creatinine, and total protein from the mid-infrared spectra of dried urine films. *Clin. Chem.* **46,** 1493–1495 (2000).
3. Heise, H. M. *et al.* Multivariate Calibration for the Determination of Analytes in Urine Using Mid-Infrared Attenuated Total Reflection Spectroscopy. *Appl. Spectrosc., AS* **55,** 434–443 (2001).
4. Pezzaniti, J. L., Jeng, T. W., McDowell, L. & Oosta, G. M. Preliminary investigation of near-infrared spectroscopic measurements of urea, creatinine, glucose, protein, and ketone in urine. *Clin. Biochem.* **34,** 239–246 (2001).
5. Oliver, K. V., Maréchal, A. & Rich, P. R. Effects of the Hydration State on the Mid-Infrared Spectra of Urea and Creatinine in Relation to Urine Analyses. *Appl. Spectrosc., AS* **70,** 983–994 (2016).
6. Bonsnes, R. W. & Taussky, H. H. On the colorimetric determination of creatinine by the Jaffe reaction. *J. Biol. Chem.* **158,** 581–591 (1945).
7. Guerra, A. *et al.* A Simple Quantitative Test for Screening Cystinuria. *Laboratory Medicine* **33,** 214–217 (2002).
